# Factors Associated with Perforated Appendicitis in Elderly Patients in a Tertiary Care Hospital

**DOI:** 10.1155/2015/847681

**Published:** 2015-08-24

**Authors:** Siripong Sirikurnpiboon, Suparat Amornpornchareon

**Affiliations:** Department of Surgery, Rajavithi Hospital, College of Medicine, Rangsit University, Phayathai Road, Rajathewee, Bangkok 10400, Thailand

## Abstract

*Background*. The incidence of perforated appendicitis in elderly patients is high and carries increased morbidity and mortality rates. The aim of this study was to identify risk factors of perforation in elderly patients who presented with clinical of acute appendicitis. *Methods*. This was a retrospective study, reviewing medical records of patients over the age of 60 years who had a confirmed diagnosis of acute appendicitis. Patients were classified into two groups: those with perforated appendicitis and those with nonperforated appendicitis. Demographic data, clinical presentations, and laboratory analysis were compared. *Results*. Of the 206 acute appendicitis patients over the age of 60 years, perforated appendicitis was found in 106 (50%) patients. The four factors which predicted appendiceal rupture were as follows: male; duration of pain in preadmission period; fever (>38°C); and anorexia. The overall complication rate was 34% in the perforation group and 12.6% in the nonperforation group. *Conclusions*. The incidence of perforated appendicitis in elderly patients was higher in males and those who had certain clinical features such as fever and anorexia. Duration of pain in the preadmission period was also an important factor in appendiceal rupture. Early diagnosis may decrease the incidence of perforated appendicitis in elderly patients.

## 1. Introduction

Acute appendicitis is the most common surgical disease, with an incidence of about 100 per 100,000. The life-time risk of developing appendicitis is 8.6% for males and 6.7% for females [1, 2], with 90% found in children and young adults and 10% in patients over 60 years old [3, 4].

Diagnosis of appendicitis is made mainly by history and physical examination, and laboratory study and radiologic investigation are helpful in equivocal cases. Clinical presentation has overall sensitivity and specificity of 45–81% and 36–53% [5], respectively. The possible cause is variation of appendix [6]. With regard to laboratory study, an increase in white blood cell count (WBC), predominance of polymorphonuclear leukocytes (PMN), and increased C-reactive protein (CRP) levels were associated with the risk and severity of complications in appendicitis [7]. With elderly patients, the diagnosis is more difficult, and this can lead to higher mortality and morbidity rates than in the general population. This study aimed to analyse factors associated with rupture in elderly patients.

## 2. Materials and Methods

This was a retrospective study of medical records which were searched for ICD-10: K35 diagnosis codes from January 2010 to December 2014. The inclusion criteria were patients who (1) had diagnosis of acute appendicitis; (2) who were aged more than 60 years; (3) who had undergone operation in Rajvithi Hospital; and (4) whose pathological results had confirmed appendicitis. Patients who had undergone appendectomy inadvertently or whose type of appendicitis (acute appendicitis or perforated appendicitis) could not be identified from medical records or pathological reports were excluded. Each case underwent open appendectomy, and drains were placed in all patients in the perforated group. Data collected included demographic data, clinical presentation, duration of pain in the preadmission period, and laboratory analysis. Statistical analysis was performed using univariate and multivariate logistic regression with SPSS version 17.0.

## 3. Results

Appendectomies were performed from 1 January 2010 to 31 December 2014 on 206 patients who were all more than 60 years old. Of these cases, 78 were males (37.9%) and 128 were females (62.1%). The mean age was 68.98 ± 7.08 years (60–91 years), and the mean BMI was 23.86 ± 3.76 (16.4–37.0). Half (103) of the appendectomies were perforated, and half (103) were nonperforated.

A total of 125 patients (60.7%) had comorbidity such as diabetes mellitus, hypertension, chronic kidney disease, chronic liver disease, cardiovascular disease, congestive heart failure, and COPD, and 71 patients had more than one comorbidity. A comparison of the basic characteristics of the groups is shown in Table 1. It was found that perforated appendicitis was associated with male sex, living in urban areas, and living alone.

With regard to clinical presentation, most patients with abdominal pain had other symptoms such as nausea, vomiting, anorexia, migratory pain from the periumbilicus to the right iliac fossa, and fever ≥38°C. Physical examination showed tenderness at the right iliac fossa, and laboratory data revealed an increase in WBC and PMN predominance. Imaging studies were done by CT (computerized tomography) scan or US (ultrasonography), and 2 patients in the perforated group and 1 in the nonperforated group underwent both. In the perforation group, the mean time to imaging was 8.53 hours (1–24 hours) while in the nonperforated group it was 5.33 hours (2–12 hours). The clinical data of the two groups are compared in Table 2. Clinical presentation data showed that anorexia, fever of more than 38°C, and time to imaging were significantly associated with perforated appendicitis. The overall median duration of pain in the preadmission period was 24 hours (2–240 hours). Most of the patients came to the hospital 24 hours after the onset of abdominal pain. Of these, 90 (87.4%) had perforated appendicitis and 66 (64.1%) had acute appendicitis. The study showed there were statistically significant differences between the two groups. The overall median duration of pain to performance of operation was 28.5 hours (4–241.5 hours); in the perforated group the mean duration was 50 hours and in the nonperforated group it was 27 hours (*p* < 0.01), and this was a statistically significant difference. Patients who underwent imaging more than 6 hours after arriving at the hospital had a significantly higher risk of perforation. Details are shown in Table 3.

With regard to intraoperative result, 6 patients in perforated group had conversion operations: 2 to right hemicolectomy and 4 patients to ileocecectomy. Univariate analysis showed that the factors associated with perforated appendicitis were male sex, fever ≥38°C, anorexia, duration of pain in the preadmission period, and duration of pain to performance of operation. Multivariate analysis revealed that the factors significantly associated with perforated appendicitis were male sex (OR = 2.36, 95% CI, 1.25–4.44), fever ≥38°C (OR = 2.17, 95% CI, 1.10–4.27), anorexia (OR = 1.92, 95% CI, 1.03–3.57), and duration of pain in the preadmission period (OR = 1.02, 95% CI, 1.01–1.04). Details are shown in Table 4.

The total number of complications was 34 (33%) in the perforated appendicitis group compared with 13 (12%) in the acute appendicitis patients (*p* < 0.001). Significant complications were pneumonia (*p* = 0.046) and surgical wound infection (*p* = 0.001). Median length of hospital stay in the perforation group was 8 days (3–48 days) and 4 days (2–136 days) in the nonperforation group, and this was statistically significant (*p* < 0.001). Of the 103 patients in the perforated appendicitis group, there were 92 cases (89.3%) of complete recovery and two mortalities (1.9%): one patient died from septic shock 10 days after the onset of abdominal pain due to delayed diagnosis, and the other one died from congestive heart failure due to multiple comorbidities and underlying valvular heart disease. In contrast, complete recovery was observed in all nonperforated patients, and there were no mortalities. A comparison of morbidity and mortality in the two groups is shown in Table 5.

An analysis of scores for predicting ruptured appendicitis is shown in Table 6. Validation scores using cut-off value 6 in this data showed sensitivity of 56% with specificity of 83% and accuracy of 69.4% as shown in Table 7.

## 4. Discussion

The incidence of acute appendicitis in elderly patients aged more than 60 years was about 5–10% [3, 8] with good postoperative outcome after appendectomy, but, in the case of perforated appendicitis, there were instances of mortality and higher rates of morbidity postoperatively. The incidence of perforated appendicitis was 32%–72% [9–14] mostly due to delayed diagnosis caused by equivocal history and physical examination [14–17]. In the present study, perforated appendicitis was found in 50% of cases which is comparable to the findings of previous research. The risk factors associated with perforated appendicitis were male sex, fever ≥38°C, anorexia, and duration of pain in the preadmission period.

In relation to risk factors, this research found that being of male sex was significantly related to perforation, and this is in line with the results of previous reports [18–20]. A possible explanation for this is elderly males' culture of reluctance to go to hospital, as found in a report by Sheu et al. [18].

With regard to social factors, living in metropolitan areas and living alone were risks for delaying seeking medical services. The author did not attempt to delve into this factor in detail, but possible explanations are changes in family structure, an increase in living away from one's family, and less real social participation.

With regard to clinical presentation, fever ≥38°C and anorexia were factors affecting the likelihood of having a perforated appendix. Previous studies have shown the same significance of fever [18, 21, 22]. A recent report by Shimizu et al. [23] confirmed the relationship between severity of fever and appendicitis and proposed that the neutrophil to lymphocyte ratio (NLR) was useful for predicting the severity of inflammation because pooled neutrophils in bone marrow are able to respond more rapidly to infectious disease compared to acute inflammation-related proteins that are produced by the liver such as C-reactive protein. In relation to the Alvarado score, the mean in the perforation group was 7.58 ± 1.49 and 7.29 ± 1.36 in the nonperforation group. An Alvarado score of more than 7 had sensitivity and specificity for diagnosing appendicitis, but high Alvarado scores did not correlate with severity of disease and could not discriminate between perforated and acute appendicitis [24]. In this study, the median duration of pain in the preadmission period in the perforated appendicitis patients was 48 hours. The results confirmed the findings of previous reports about the risk of perforation from delaying seeking medical attention [8, 11, 13, 21, 25–28]. A recent study by Augustin et al. [29] showed that the risk of perforation increased 36 hours after onset of pain. Similarly, in a report about another age group, Singh et al. [30] showed a significant association between perforated appendicitis in pediatric patients and a duration of pain to admission of longer than 72 hours. With regard to time to imaging, this was significantly longer in the perforated group compared to the nonperforated one. Generally, clinical examination is more important than investigation, but the latter can be helpful where the clinical picture is equivocal in patients of extreme age. A study from Gardner et al. [31] showed imaging influenced elderly patient management in 36% of cases and affected diagnosis; however, the impact of duration from admission to operation is still a controversial issue. A report by Eko et al. [32] suggested that it should not exceed 18 hours in order to reduce postoperative morbidities and length of stay. Busch et al. [33] showed that a delay of more than 12 hours was associated with a significant increase in the rates of perforation. In contrast, another study did not show any significant difference: Partelli et al. [34] reported that delays in performing appendicitis operations did not increase postoperative complications. Similarly, Abou-Nukta et al. [17] reported that delaying appendectomy by 12–24 hours after presentation did not significantly increase the rate of perforation, operative times, or length of stay; furthermore, a recent report by Teixeira et al. [35] found that delays in the time from diagnosis to operation did not increase perforation rates.

The mortality rate of perforated appendicitis in elderly patients was about 2.3% to 10% and most commonly correlated with infection and underlying comorbid disease [8, 11–13]. In our study, there were 2 deaths (1.9%) from sepsis and underlying comorbid disease, similar to the results of other studies.

One limitation of this study was that it was a retrospective one, so that we were unable to collect some significant data which could possibly have affected the outcomes, such as patient race, economic status, type of appendicitis, and CRP level.

## 5. Conclusion

Male sex, fever ≥38°C, anorexia, and duration of pain in the preadmission period were the significant factors associated with perforated appendicitis in elderly patients in this study.

## Figures and Tables

**Table 1:** Comparison of patients' characteristics in the perforated and nonperforated appendicitis groups.

	Perforated appendicitis (*n* = 103)	Nonperforated appendicitis (*n* = 103)	*p* value
Age (mean ± SD) (years)	68.8 ± 7.4	69.2 ± 6.8	0.989
Male sex	49 (47.6)	29 (28.2)	0.004^*∗*^
Address			<0.001^*∗*^
Urban	74 (71.9)	36 (35.0)	
Suburb	29 (28.1)	67 (65.0)	
Living status			<0.001^*∗*^
With family	82 (86.3)	30 (29.1)	
Living alone	13 (13.7)	63 (70.9)	
Underlying disease			0.770
Diabetes mellitus	32 (31.1)	25 (24.3)	0.276
Hypertension	56 (54.4)	54 (52.4)	0.780
Myocardial infarction	10 (9.7)	11 (10.7)	0.818
Congestive heart failure	1 (1.0)	2 (1.9)	1.000
Chronic kidney disease	8 (7.8)	5 (4.9)	0.390
Chronic liver disease	0 (0)	2 (1.9)	0.498
COPD	3 (2.9)	3 (2.9)	1.000
ASA classification			0.218
I	11 (10.7)	8 (7.8)	
II	76 (73.8)	86 (83.5)	
III	16 (15.5)	9 (8.7)	
BMI (mean ± SD) (Kg)	23.8 ± 4.2	23.9 ± 3.3	0.525

*∗*: value < 0.05 is statistically significant.

**Table 2:** Comparison of clinical presentation in the two groups.

Clinical presentation	Perforated appendicitis (*n* = 103) (%)	Acute appendicitis (*n* = 103) (%)	*p* value
Nausea and/or vomiting	67 (65)	60 (58.3)	0.316
Anorexia	65 (63.1)	50 (48.5)	0.035^*∗*^
Migratory pain	60 (58.3)	58 (56.3)	0.778
Fever > 38°C	44 (42.7)	26 (25.2)	0.008^*∗*^
RLQ tenderness	102 (99)	103 (100)	1.000
Rebound tenderness	91 (88.3)	83 (80.6)	0.124
WBC > 10 × 10^9^ cell/L	87 (84.5)	89 (86.4)	0.693
Neutrophil > 75%	74 (71.8)	83 (80.6)	0.141
Alvarado score (mean ± SD)	7.58 ± 1.49	7.29 ± 1.36	0.199
Imaging study	31 (48.4)	33 (51.6)	0.763
Computerized tomography	22 (21.4)	22 (21.4)	1.000
Acute appendicitis	4 (18.2)	21 (95.5)	<0.001^*∗*^
Ruptured appendicitis	18 (81.8)	1 (4.5)	
Ultrasonography	11 (10.7)	12 (11.7)	0.825
Acute appendicitis	6 (54.5)	12 (100.0)	0.024^*∗*^
Ruptured appendicitis	5 (45.5)	0 (0.0)	
Time to imaging (mean ± SD)	8.53 ± 3.57	5.33 ± 2.33	<0.001^*∗*^

*∗*: value < 0.05 is statistically significant.

**Table 3:** Duration of time in perforated and acute appendicitis groups.

	Perforated appendicitis *n* = 103	Acute appendicitis (*n* = 103)	*p* value
Duration of pain in admission period	48 (6–240)	24 (2–96)	
<24 hours	13 (12.6%)	37 (35.9%)	<0.001^*∗*^
≥24 hours	90 (87.4%)	66 (64.1%)	
Duration from pain to operation	50 (8–241)	27 (4–104)	
<24 hours	11 (10.7%)	35 (34.0%)	<0.001^*∗*^
≥24 hours	37 (89.3%)	46 (66.1%)	
Duration from admission to operation	6 (1–8)	10 (9–12)	
>8 hours	16 (15.5%)	12 (11.7%)	0.416
≤8 hours	87 (84.5%)	91 (88.3%)	
Duration from arrival to imaging	8 (1–24)	6 (2–12)	
>6 hours	78 (75.7)	30 (29.1)	<0.001^*∗*^
≤6 hours	25 (24.3)	73 (70.9)	

Value s are represented as numbers (percentages) and median (minimum-maximum).

*∗*: value < 0.05 is statistically significant.

**Table 4:** Factors associated with perforated appendicitis by multivariate analysis.

Factor	Adjusted odds ratio	95% confidence interval	*p* value
Male sex	2.47	1.31–4.63	0.008
Fever > 38°C	1.97	1.03–3.78	0.024
Anorexia	1.90	1.03–3.52	0.040
Duration of pain in preadmission period	4.21	2.22–7.98	<0.001

**Table 5:** Outcomes, complications, and length of hospital stay.

Results	Perforated appendicitis (*n* = 103)	Acute appendicitis (*n* = 103)	*p* value
Operation conversion *n* (%)	8 (7.8)	0	0.003^*∗*^
Complication *n* (%)	34 (33)	13 (12.6)	<0.001^*∗*^
Pneumonia	16 (15.5)	7 (6.8)	0.046^*∗*^
Respiratory failure	4 (3.9)	1 (1.0)	0.174
Gastrointestinal bleeding	2 (1.9)	0 (0)	0.498
Surgical wound infection	19 (18.4)	4 (3.9)	0.001^*∗*^
Length of hospital stay			
Median (min-max)	8 (3–48)	4 (2–136)	<0.001
Discharge status *n* (%)			0.005^*∗*^
Complete recovery	92 (89.3)	102 (99)	
Morbidity	9 (8.7)	1 (1)	
Death	2 (1.9)	0 (0)	

*∗*: value < 0.05 is statistically significant.

**Table 6:** Scores for predicting ruptured appendicitis in elderly patients.

Factor	Adjusted OR	Score
Male	2.47	2
Fever (*T* > 38°C)	1.97	2
Anorexia	1.90	2
Pain > 24 hrs	4.21	4
Total score		10

**Table 7:** Validation scores for predicting ruptured appendicitis.

	Perforation		Total	Sensitivity	Specificity	PPV	NPV	Accuracy
	Yes	No						
Score								
≥6	58	18	76	58/103 = 56%	85/103 = 83%	58/7 = 76%	85/130 = 65%	143/206 = 69.4%
<6	45	85	130					
Total	103	103						
